# Prognostic Role of ^18^F-FDG PET/CT in Oligometastatic Non-Small Cell Lung Cancer: Preliminary Results from Single Center

**DOI:** 10.3390/cancers18121880

**Published:** 2026-06-09

**Authors:** Artur Bandura, Monika Mierzejewska, Wojciech Cytawa, Rafał Dziadziuszko

**Affiliations:** 1 Department of Oncology and Radiotherapy, Medical University of Gdańsk, 80-210 Gdańsk, Poland; 2 Department of Nuclear Medicine, Medical University of Gdańsk, 80-210 Gdańsk, Poland

**Keywords:** oligometastatic, non-small cell lung cancer, 18F-fluorodeoxyglucose positron emission tomography/computed tomography, radiotherapy, metabolic tumor volume, total lesion glycolysis

## Abstract

**Background:** Oligometastatic non-small cell lung cancer (OMD NSCLC) represents a distinct clinical state with limited metastatic spread, where local ablative therapies (LAT) combined with systemic treatment may improve outcomes. Accurate staging and prognostic assessment are critical, with ^18^F-FDG PET/CT emerging as a valuable tool for detecting metabolically active tumor burden. **Results:** We retrospectively analyzed 38 patients with synchronous OMD NSCLC who received radiotherapy. All patients underwent ^18^F-PET/CT and brain imaging for staging. Semi-quantitative ^18^F-PET/CT parameters, including metabolic tumor volume (MTV) and total lesion glycolysis (TLG) of primary tumor and metastatic lesions, were measured and associated with progression-free survival (PFS) and overall survival (OS) using Cox proportional hazard models. The median PFS and OS were 8.28 and 21.62 months, respectively. Higher MTV and TLG values of metastases were significantly associated with shorter PFS and OS (*p* < 0.01). In multivariable analysis, TLG of metastases above the median remained an independent predictor of worse PFS (HR = 2.78, *p* = 0.031) and OS (HR = 3.12, *p* = 0.024), alongside clinical factors such as ECOG performance status 2 and having multiple metastases. The metabolic parameters of the primary tumor did not predict survival outcomes. **Conclusions:** ^18^F-PET/CT-derived metabolic parameters, particularly the TLG of metastatic lesions, may provide significant prognostic information in oligometastatic NSCLC beyond clinical variables. These findings may support the integration of ^18^F-PET/CT metrics for future trials involving patients treated with LAT in oligometastatic NSCLC.

## Simple Summary

We analyzed patients with a limited number of lung cancer metastases (oligometastatic disease) who were treated with local therapies. Using PET/CT scans, we measured the metabolic activity of the cancer in both the primary tumor and metastatic lesions. We found that patients with a higher metabolic volume of their metastases had significantly shorter survival, even after accounting for other clinical factors. In contrast, the metabolic parameters of primary tumors were not associated with outcomes. Overall, PET/CT, particularly the measurement of metabolic activity in metastases, provides useful prognostic information and may help guide treatment decisions in this group of patients.

## 1. Introduction

Stage IV non-small cell lung cancer (NSCLC) is associated with a poor prognosis, with a median overall survival (OS) typically ranging from 8 to 16 months, depending on the treatment approach and molecular profile of the tumor [1]. In locally advanced (stage III) NSCLC, the expected OS ranges from approximately 15 to 36 months and is influenced by factors such as tumor resectability, extent of nodal involvement, and the application of multimodal therapy, including chemoradiotherapy and consolidation immunotherapy [2]. In 1995, Weichselbaum and Hellman first postulated the concept of an oligometastatic state, a transitional clinical stage characterized by limited metastatic spread, in which patients may benefit from aggressive local ablative therapies (LATs) with curative intent, despite the disseminated cancer process [3,4]. Oligometastatic disease (OMD) in NSCLC is increasingly recognized as a distinct biological state characterized by limited metastatic spread, typically defined as ≤5 lesions in ≤3 organs [5,6,7,8]. This intermediate state offers the potential for long-term survival or cure when aggressive local therapies are combined with systemic treatments [9]. Advances in systemic therapy, particularly immune checkpoint inhibitors, have improved outcomes in metastatic NSCLC, thereby reinforcing the importance of accurate staging and disease characterization. Current real-world results show that the survival of OMD could be similar to that of stage III [10,11].

Conventional imaging modalities, such as contrast-enhanced CT and MRI, often fail to detect subclinical nodal or distant metastases. In contrast, ^18^F-fluorodeoxyglucose positron emission tomography/computed tomography (^18^F-FDG PET/CT) enhances diagnostic accuracy by detecting metabolically active tumor lesions [1,12,13], leading to better selection of patients suitable for curative-intent local therapies [8]. According to the guidelines, patients should be staged with PET/CT and adequate brain imaging (CT with contrast enhancement or brain MRI, which is the preferred option) to confirm the oligometastatic state [5,6,7]. Moreover, ^18^F-PET/CT-derived quantitative biomarkers, particularly metabolic tumor volume (MTV) and total lesion glycolysis (TLG), were shown to associate with progression-free survival (PFS) and OS in NSCLC [14]. These findings support the routine integration of ^18^F-PET/CT into the management of oligometastatic NSCLC. Another interesting application of ^18^F-PET/CT is its potential to predict histopathological subtypes, the presence of EGFR mutations, and the level of PD-L1 expression [15,16,17,18,19].

The aim of this study is to evaluate ^18^F-PET/CT parameters as prognostic tools in the management of oligometastatic NSCLC treated with LAT. We present results based on patients treated in a single center and discuss them with the current literature to provide a broader view of the utility of ^18^F-PET/CT in OMD NSCLC.

## 2. Materials and Methods

We retrospectively reviewed the records of patients treated with radiation therapy for synchronous oligometastatic disease at the single academic center, between January 2016 and December 2024. All consecutive patients were screened for inclusion in the analysis. Eligible patients were required to meet all of the following inclusion criteria: (1) availability of PET/CT imaging for staging purposes together with brain imaging (MRI or CT); (2) diagnosis of synchronous NSCLC (defined as ≤5 lesions in ≤3 organs); (3) treatment of at least one lesion with radiotherapy; and (4) local treatment of all disease sites, including both primary and metastatic lesions, using local modalities such as radiotherapy or surgery. The exclusion criteria were: (1) lack of local treatment for all disease sites; (2) use of palliative radiotherapy; and (3) absence of pre-treatment PET/CT imaging prior to qualification for therapy.

The analysis was based on 38 patients. Survival time was calculated from the day of treatment start until progression of disease (PFS) or death (OS). Progression was defined as any of the following: radiological progression determined by the institutional radiologist (contrast-enhanced CT performed routinely every 3 months during the first 2 years and every 6 months thereafter), clinical progression, or death. The reverse Kaplan–Meier method was used to estimate the median follow-up time, allowing for assessment of the maturity of the overall survival data. The semi-quantitative parameters MTV and TLG (defined as the multiplication of MTV by SUVmean [SUV, standardized uptake value, parameter normalized to body weight, SUV-bw, and reported as SUV × cm^3^]) were calculated for each ^18^F-FDG PET/CT by two researchers (A.B. and M.M.) using the syngo.via software (version VB60A, Siemens Healthineers, Erlangen, Germany). Researchers performed MTV and TLG measurements on the PET/CT scans jointly and were blinded to the survival outcomes during the analysis. Each metastatic site (including mediastinal lymph nodes) was considered a separate volume of interest (VOI), when feasible, and a fixed 40% relative threshold of the tumor SUV_max_ was used to semi-automatically draw the VOI of each lesion, as previously described [20,21]. Briefly, first a larger spherical VOI was manually delineated to cover the entire lesion, then a relative 40% SUV max threshold was set to obtain the MTV and consequently TLG (by multiplying MTV by SUVmean). When the volume of hilar lymph nodes could not be discriminated from the primary lesion because of merging into one mass, it was counted as metabolic tumor volume and total lesion glycolysis of the primary tumor (MTV_prim_ and TLG_prim_, respectively). On the other hand, MTV_mets_ and TLG_mets_ were obtained by summing up metabolic volumes and glycolysis of all metastatic lesions considered to be related to cancer, except for brain metastases, similar to other authors [22]. Central nervous system (CNS) lesions were not included because of the high ^18^F -FDG avidity of the healthy brain parenchyma. Moreover, at our center, ^18^F-FDG PET/CT is routinely performed from the base of the skull to the mid-thigh, and other modalities (MRI or CT with contrast enhancement) are used for CNS staging. We calculated SUV_max_, TLG, and MTV separately for the primary and metastatic sites, as well as for the sum of all lesions, and investigated the relationship between PET/CT findings and the survival in patients treated with radiotherapy as LAT for OMD. The radiotherapy details were described in our previous publication that reported the treatment outcomes of patients with synchronous and metachronous lung cancer [10]. Clinical factors known in the literature for their relevance in OMD NSCLC (ECOG performance status, number of metastases, exposure to systemic treatment, and brain involvement) were analyzed [11,23].

Univariable and multivariable analyses were performed using Cox proportional hazard models. Variables that passed the univariable screening (threshold < 0.15) were entered into a backward stepwise selection algorithm based on likelihood ratio tests. Statistical significance was defined as *p* ≤ 0.05 throughout the study. Statistical analysis was performed using the IBM SPSS Statistics (version 29.0.0). The Local Bioethics Committee for Scientific Research at the Medical University of Gdańsk approved this study (approval no. NKBBN/575/2021).

## 3. Results

**Figure 1:**
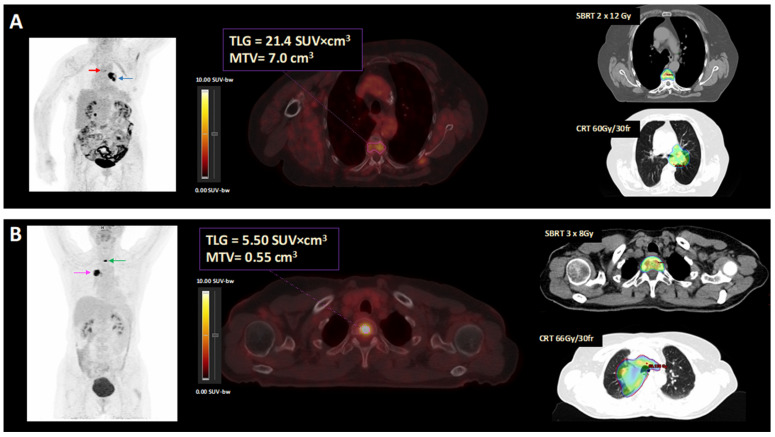
The figure illustrates two cases of patients with synchronous oligometastatic cancer, including representative PET-CT images. The upper panel (**A**) represents a female patient with a primary tumor in the left lung with nodal involvement (blue arrow) and a single distant metastatic lesion (red arrow) in the vertebral body (Th6, confirmed by MRI with contrast enhancement). Both metabolic parameters (TLG and MTV) of the metastatic lesion in the bone exceeded the median values observed in our study. The patient underwent concomitant radiochemotherapy (60 Gy/30 fr) and subsequently received stereotactic radiotherapy (24 Gy/2 fr) for the metastatic lesion in the vertebra. The progression-free survival (PFS) was 4.5 months, and the overall survival (OS) was 10.4 months. The lower panel (**B**) shows a female patient with a primary tumor in the right lung with nodal involvement (pink arrow) and a single distant metastatic lesion in the vertebral body of Th2 (green arrow). The patient was treated with concurrent chemoradiotherapy (66 Gy/30 fr) to the primary tumor and stereotactic radiotherapy (24 Gy/3 fr) to the metastatic site. The MTV and TLG were below the median values observed in our cohort, and PFS and OS were 13.7 and 44.6 months, respectively.

**Figure 2:**
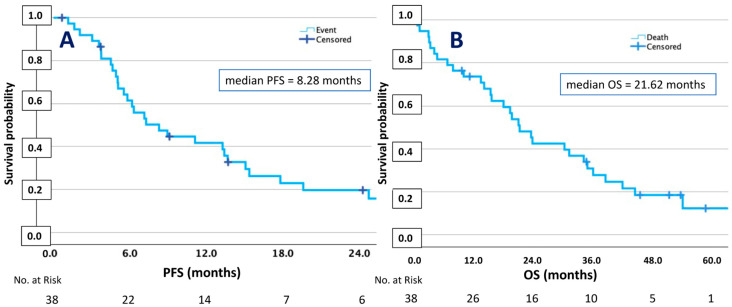
Probability of the progression-free survival (**A**) and overall survival (**B**) in all of the patients with OMD NSCLC.

The study included 38 patients with a median age of 69 years (range 50–85). Median follow up time was 53.8 months. There was a slight female predominance (52.6%). Most patients had an ECOG performance status of 1 (55.3%), while 21.1% had ECOG 0, and 18.4% had ECOG 2. Adenocarcinoma was the predominant histological subtype (60.5%), followed by squamous-cell carcinoma (31.6%). Most patients presented with a single metastatic lesion (76.3%) and involvement of only one organ (89.5%), whereas a smaller proportion had two or three metastases or involvement of two organs. The study population and baseline patient characteristics are shown in Table 1. Representative PET/CT scans of patients with metabolic parameters above and below the median, along with the applied treatments, are shown in Figure 1. Table summarizing metabolic parameters of the primary tumors and metastases is added in Supplementary Table S1. The median PFS and OS for all patients were 8.28 (95%CI: 4.43–12.13) and 21.62 months (95%CI: 16.47–26.76), respectively (Figure 2A,B). Univariable and multivariable analysis both for PFS and OS is shown in Table 2 and Table 3.

### 3.1. Progression Free Survival (PFS)

**Figure 3:**
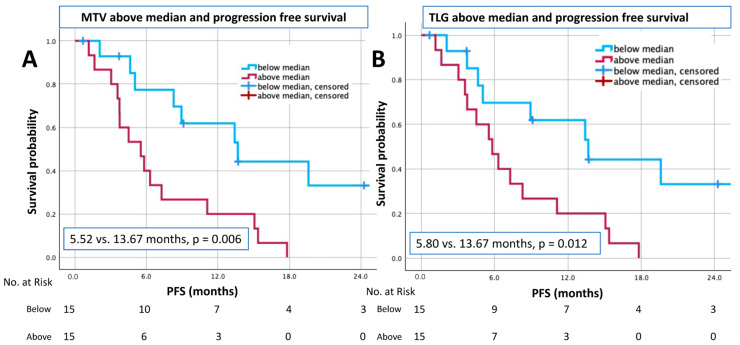
Probability of the progression-free survival in patients with MTV_mets_ (**A**) and TLG_mets_ (**B**) below and above median.

In the univariable analysis, several PET/CT-derived and clinical variables were significantly associated with PFS (Table 2). Patients with MTV_mets_ above the median (2.19 cm^3^) had a significantly higher risk of progression (*p* = 0.009; HR = 3.35; 95% CI: 1.35–8.32) and experienced shorter PFS than those with MTV below the median (5.52 vs. 13.67 months, respectively; *p* = 0.006, Figure 3A). Similarly, total lesion glycolysis (TLG) of metastases above the median value of 11.01 SUV × cm^3^ was also associated with worse PFS (5.80 vs. 13.67 months, respectively; *p* = 0.012, Figure 3B) and higher risk of progression (HR = 3.04; 95% CI: 1.22–7.56). In contrast, none of the metabolic parameters of the primary lesion (SUV_max_, MTV_prim_, and TLG_prim_) were prognostic for PFS. In addition, the sum of the metabolic volumes of all cancer lesions (both primary and metastatic) was not associated with PFS. Among the clinical factors, ECOG performance status of 2 was a strong negative prognostic indicator (*p* = 0.001; HR = 4.78; 95% CI: 1.85–12.39), as was having more than one metastatic lesion (*p* = 0.010; HR = 3.27; 95% CI: 1.32–8.11). Not receiving systemic treatment was also associated with shorter PFS (*p* = 0.035; HR = 2.20; 95% CI: 1.06–4.57). Brain involvement was not significantly associated with PFS.

In the multivariable analysis, TLG of metastases above the median remained independently associated with inferior PFS (*p* = 0.031; HR = 2.78; 95% CI: 1.10–7.05). In addition, ECOG performance status 2 (vs. 0–1) was independently predictive of shorter PFS (*p* = 0.014; HR = 3.64; 95% CI: 1.30–10.24), as was having more than one metastasis (*p* = 0.007; HR = 6.07; 95% CI: 1.65–22.31).

### 3.2. Overall Survival (OS)

**Figure 4:**
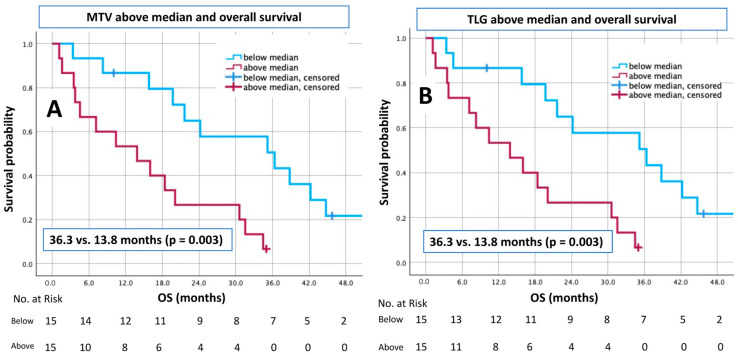
Probability of the overall survival in patients with MTV_mets_ (**A**) and TLG_mets_ (**B**) below and above median.

Lower values of MTV_mets_ and TLG_mets_ were associated with longer OS, with a median OS of 36.3 vs. 13.8 months (*p* = 0.003), showing the same result for both parameters (Figure 4A,B). Univariable analysis also identified several predictors of OS (Table 3). Patients with MTV of metastases above the median had significantly shorter OS (*p* = 0.006; HR = 3.94; 95% CI: 1.50–10.39), and those with high TLG had similarly poor outcomes (*p* = 0.006; HR = 3.88; 95% CI: 1.47–10.23). ECOG performance status of 2 was the most powerful negative predictor in the model (*p* < 0.001; HR = 6.49; 95% CI: 2.30–18.30), while absence of systemic therapy also predicted worse survival (*p* = 0.048; HR = 2.14; 95% CI: 1.01–4.53). As for PFS, none of the metabolic parameters (SUV_max_, MTV, and TLG) of the primary tumor or the sum of all metabolic volumes of cancer lesions were prognostic for OS. Brain involvement was not significantly associated with OS.

In the multivariable analysis, TLG of metastases above the median remained a significant independent prognostic factor for OS (*p* = 0.024; HR = 3.12; 95% CI: 1.16–8.35). ECOG performance status of 2 also retained its strong association with poorer survival (*p* = 0.006; HR = 4.59; 95% CI: 1.54–13.66).

## 4. Discussion

Current guidelines for the diagnosis and treatment of NSCLC recommend ^18^F-FDG PET/CT as the imaging modality of choice for accurate staging [1,24]. This modality can distinguish patients with local disease from those who already have metastatic spread, but is not detectable in conventional modalities [14]. According to the ESTRO/ASTRO and EORTC statements, patients should undergo proper staging with ^18^F-FDG PET/CT and brain imaging [5,6,7]. Those with no more than five metastases in no more than three organs and with all lesions amenable to local treatment are typically qualified as having oligometastatic NSCLC [5,7,25]. The above was performed in all patients included in the analysis.

Although the Tumor-Node-Metastasis (TNM) system is considered the most important tool for estimating prognosis and guiding treatment decisions, quantitative parameters, such as FDG uptake, could become important indicators of survival. Brose et al. conducted a multicenter retrospective study evaluating that MTV derived from baseline ^18^F-FDG PET/CT adds prognostic value beyond traditional TNM staging in locally advanced (stage III) NSCLC [26]. The analysis included 235 patients from the PET-Plan trial and a clinical registry cohort who were treated mainly with chemoradiotherapy. In the multivariable Cox regression analysis, MTV was independently associated with worse OS, even after adjustment for TNM stage and clinical variables. Similar results were obtained in a secondary analysis of the ACRIN 6668/RTOG 0235 trial, where pre-treatment total MTV on PET was associated with OS in patients with inoperable stage III NSCLC treated with definitive chemoradiation [27]. Among the 230 evaluable patients, those with total MTV above the median value of 32 mL had significantly worse median OS compared to those with lower MTV (14.8 vs. 29.7 months, *p* < 0.001). A higher MTV was independently associated with inferior survival when analyzed as a continuous variable. Importantly, a significant interaction between radiation dose and MTV was observed, indicating that the adverse prognostic impact of a high tumor volume decreased with higher radiation doses. In patients with a large total MTV, doses > 60 Gy were associated with improved survival. The authors concluded that an elevated pre-treatment MTV is a strong negative prognostic factor and that adequate radiation dosing may mitigate its impact. Also several studies confirmed prognostic role of PET/CT [28,29,30,31].

A recent meta-analysis by Ling et al. included 13 studies that evaluated the prognostic value of baseline ^18^F-FDG PET/CT metabolic parameters in patients with advanced or metastatic NSCLC treated with immune checkpoint inhibitors. High baseline MTV and TLG were significantly associated with worse OS: HR 2.10 (95% CI 1.57–2.82) and HR 1.58 (95% CI 1.03–2.44), respectively. In contrast, SUV_max_ and SUV_mean_ were not reliable prognostic markers [32]. The authors concluded that baseline MTV and TLG appear to be the most robust PET-derived prognostic parameters in NSCLC patients receiving immunotherapy, although heterogeneity and the lack of standardized thresholds remain limitations. Importantly, this analysis did not include patients with OMD. More recent evidence from a meta-analysis with sensitivity analyses confirms that ^18^F-PET/CT-derived MTV is a prognostic biomarker for immunotherapy in NSCLC [33].

Our findings that the metabolic parameters of the tumor burden could be prognostic for survival are consistent with previous results reported by other groups. Chin et al. conducted a retrospective cohort study of 55 patients with oligometastatic NSCLC who underwent high-dose radiotherapy of all sites of active disease. Their analysis showed that greater MTV and TLG were predictive of shorter OS (HR, 2.42 and 2.14, respectively); however, they did not report PFS [34]. Multicenter retrospective study by Ogliari et al. evaluated the prognostic value of baseline MTV and TLG in 105 patients with synchronous oligometastatic NSCLC treated with first-line immunotherapy-based regimens [22]. A lower MTV was significantly associated with longer progression-free survival (14.7 vs. 7.6 months, *p* = 0.03), whereas TLG showed a similar but weaker trend. Neither MTV nor TLG significantly impacted OS. Although the authors reported interesting results with a novel systemic treatment (immunotherapy, chemoimmunotherapy), they were not statistically significant in the analysis of the subgroup treated with LAT (n = 30). Our study provides evidence that TLG of metastatic lesions is significant for both OS and PFS in patients with oligometastatic NSCLC treated with LAT to all disease sites. Other studies that used ^18^F-PET/CT to assess prognosis in oligometastatic disease consisted of various origins or certain locations of metastases (ex. lung, liver) [35,36,37,38]. Whether TLG and MTV are prognostic factors for OMD NSCLC treated with a combination of immunotherapy and radiotherapy remains an open question.

Mazolla et al. assessed whether ^18^F-FDG PET/CT parameters could predict early response after stereotactic ablative radiotherapy for lung oligometastases [37]. They analyzed 70 lesions from 50 patients (mixed origin, not only NSCLC) and found that MTV and TLG were not predictive of local failure or distant progression. However, pre-treatment SUVmax < 5 and SUVmean < 3.5 were significantly associated with complete response at 6 months after SABR (SUVmax: *p* = 0.001, AUC = 0.90). The authors concluded that baseline SUV parameters may help identify patients who are more likely to achieve a complete response after stereotactic radiotherapy and could support personalized follow-up strategies.

In our study, the metabolic parameters of the primary tumor did not influence the outcome. Moreover, total MTV and TLG were not prognostic for survival because metastases constituted only a small proportion of the total tumor volume compared with the primary tumor. The lack of impact of primary tumor parameters may be related to the small sample size. However, it is most likely that prognosis is determined not by the size of the primary disease (which can be controlled by local treatment) but by the metastatic tumor load, which shows how much the disease has spread to other organs, with metastatic aggressiveness that limits survival. This idea is supported by Willman et al., who showed that distant metastasis velocity (defined as the number of new metastases per month categorized as <0.5, 0.5 to 1.5, and >1.5) has an impact on survival [39]. In addition, our previous report revealed that the time of occurrence of metastases in metachronous OMD is prognostic for survival [10]. Other metrics that may be clinically useful in OMD include brain metastasis velocity, PSA doubling time (in prostate cancer), and tumor-specific growth rate, which may provide more biologically relevant information than lesion count alone [40]. Although pretreatment PET/CT is a one-time-point assessment of disease spread, the metabolic burden of the disease before treatment may provide insight into the potential for further metastatic spread and, therefore, the prognosis of patients. A possible underlying mechanism could involve the Warburg effect [41]. This effect, reflected by ^18^F-PET/CT, is characterized by increased glucose uptake and lactate production despite the presence of oxygen (aerobic glycolysis). It is driven by oncogenic alterations and HIF-1α activation, leading to enhanced glycolysis, reduced oxidative phosphorylation, and increased angiogenesis, which in turn is associated with greater tumor aggressiveness [42,43,44]. In addition, ^18^F-FDG PET metrics (TLG and MTV) correlated with pathways related to gluconeogenesis and cell membrane synthesis [45].

Interestingly, a pilot study by Avella et al. demonstrated that TLG, but not SUV, correlates with the presence of circulating tumor cells in NSCLC patients without distant metastases; therefore, TLG could be an appropriate marker for hematogenous micrometastatic potential [46]. This is supported by the study by Shang et al., in which baseline metabolic tumor burden parameters (MTV and TLG), assessed from metastatic lymph nodes and whole-body tumor burden rather than SUVmax, were identified as significant predictive factors for brain metastasis development in patients with locally advanced NSCLC [47]. Hence, the TLG may better reflect the tumor metastatic potential than single-voxel intensity metrics (like SUVmax) [41].

In our study, patients with metastases to the CNS as the only organ affected by metastatic spread were also included in the analysis. However, only metabolic parameters of the primary lesion were estimated, without parameters such as MTV or TLG for brain metastases, as stated in the methodology section. The exclusion of brain metastasis volume represents a limitation of PET/CT-based quantitative assessment, as it may introduce bias in the evaluation of total metastatic burden. However this should not affect the overall results of the study for two reasons. First, the group in which MTV and TLG for metastases were calculated also included patients with brain metastases, however these brain lesions were not included in the calculations of MTV and TLG. Second, metastatic spread to the CNS was not statistically significant for PFS or OS in our study (Table 2 and Table 3).

Our study has several limitations. First, is the small sample size (n = 38) particularly for the multivariable Cox analyses, where the limited number of events may increase the risk of model instability. Second, multiple PET-derived parameters were evaluated, which increases the risk of chance findings due to multiplicity. Therefore, the findings should be considered exploratory and interpreted with caution, pending validation in larger cohorts. Third, the retrospective, single-center nature of the study introduces a potential selection bias, as only patients considered eligible for local ablative therapy (LAT) by the treating physicians were included. As a result, the cohort may represent a selected group of patients with more favorable disease characteristics, limiting the generalizability of the findings. Also this was a single-center assessment with possible implications related to local practice patterns. Another point is that majority of patients underwent radiotherapy alone or in combination with chemotherapy as systemic treatment; therefore, these findings should be interpreted cautiously, as contemporary treatment pathways for oligometastatic disease increasingly incorporate broader use of immunotherapy and targeted therapies, which were not routinely used in the studied cohort. Last, the relative 40% SUVmax threshold that was used in our analysis may lose some information about the tumor heterogeneity and result in underestimation of tumor volumes. On the other hand, a small lesion with low signal to background ratio can be overestimated by fixed relative threshold method. The reason why we decided to use this approach is that these methods are the most widely and best validated methods of delineating and calculating metabolic tumor parameters, while the algorithm based segmentation methods have not been systematically tested for accuracy, robustness, and repeatability [20].

Our observations are generally consistent with those of previous studies supporting the role of MTV and TLG as potential prognostic biomarkers; however, further prospective validation is warranted.

## 5. Conclusions

Despite existing guidelines, optimal patient selection for OMD treatment remains a therapeutic challenge, and a definition based only on the number of metastases (≤5 lesions) and affected organs (≤3 sites) may be suboptimal. Our exploratory study confirms previous data reported in the literature and adds to the existing body of evidence that metabolic parameters derived from ^18^F-PET/CT may have a prognostic impact on the survival of patients with oligometastatic NSCLC. We found that the TLG of metastases showing metabolic tumor burden is significant in assessing the prognosis of patients (both OS and PFS) and is as important as clinical factors, e.g., ECOG performance status. Taken together, these data indicate that ^18^F-PET/CT may contribute to prognostic assessment and treatment decision-making in oligometastatic NSCLC. However, our study is exploratory and we cannot draw conclusions about the prognostic role of ^18^F-PET/CT in the management of OMD with immunotherapy as a backbone treatment.

## Figures and Tables

**Table 1:** General characteristics of the analyzed group.

Age	Median: 69; Range: 50–85
No of patients (%)	
Sex	
Male	18 (47.4%)
Female	20 (52.6%)
ECOG	
0	8 (21.1%)
1	21 (55.3%)
2	7 (18.4%)
N/A	2 (5.3%)
Histopathology	
ACC	23 (60.5%)
SCC	12 (31.6%)
LCC	2 (5.3%)
NOS	1 (2.6%)
Number of metastases	
1	29 (76.3%)
2	6 (15.8%)
3	3 (7.9%)
Number of affected organs	
1	34 (89.5%)
2	4 (10.5%)
PD-L1 status	
<1%	10 (26.3%)
1–49%	5 (13.2%)
50%	1 (2.6%)
N/E	22 (57.9%)
Driver mutations	
None	19 (50.0%)
Non-targetable	5 (13.2%)
N/E	14 (36.8%)
Primary lesion treatment	
SBRT (55–60 Gy/5–8 fr)	10 (26.3%)
RT alone (60–66 Gy/15–33 fr)	8 (21.0%)
Concomitant radiochemotherapy (60–66 Gy/30–33 fr)	15 (39.5%)
Resection (lobectomy)	5 (13.2%)
Systemic treatment	
Platinum-based chemotherapy	19 (50.0%)
Immunotherapy	1 (2.6%)
None	18 (47.4%)
Metastases treatment (location and radiation doses)	
Lung	50–55 Gy/5 fr, 54 Gy/3 fr, 30 Gy/1 fr
Brain	16–20 Gy/1 fr, 22–27 Gy/3 fr, 30–35 Gy/5 fr
Adrenal	45–60 Gy/4–8 fr
Bone	16–24 Gy/1–3 fr
Brain involvement	
Yes	14 (36.8%)
No	24 (63.2%)
Brain metastases management	
stereotactic RT alone	9 (64.3%)
resection + stereotactic RT to the tumor bed	5 (35.7%)

ACC—adenocarcinoma, LCC—large-cell carcinoma, NOS—not otherwise specified, SBRT—stereotactic body radiotherapy, SCC—squamous-cell carcinoma, RT—radiotherapy, N/A—not available (due to the retrospective nature of the study, some data not documented in the medical records were unavailable). N/E—not evaluated; in some cases, the biopsy material was sufficient only for establishing the diagnosis but insufficient for NGS or PD-L1 testing or it was not assessed. Additionally, patients with squamous histology were not routinely assessed for driver mutations. Non-targetable—mutations for which no approved first-line targeted therapy was available at the time of diagnosis (e.g., KRAS G12C, EGFR exon 20, KRAS G12V, KRAS G13C).

**Table 2:** Univariable and multivariable analysis for the PFS.

Progression Free Survival						
	Univariable Analysis			Multivariable Analysis		
	*p*-Value	HR	95% CI	*p*-Value	HR	95% CI
**Primary Tumor**						
SUVmax above median	0.341	0.71	0.35–1.45			
MTV above median	0.457	0.76	0.37–1.56			
TLG above median	0.383	0.73	0.36–1.48			
**Metastases**						
SUVmax above median	0.408	1.42	0.62–3.29			
MTV above median	0.009	3.35	1.35–8.32			
TLG above median	0.017	3.04	1.22–7.56	0.031	2.78	1.10–7.05
**Sum of primary and metastases**						
MTV above median	0.835	1.09	0.47–2.50			
TLG above median	0.783	0.89	0.39–2.03			
**Clinical**						
ECOG (0 + 1 vs. 2)	0.001	4.78	1.85–12.39	0.014	3.64	1.30–10.24
No. of metastases (1 vs. more)	0.010	3.27	1.32–8.11	0.007	6.07	1.65–22.31
Systemic treatment (yes vs. no)	0.035	2.20	1.06–4.57			
Brain involvement (yes vs. no)	0.965	1.02	0.48–2.15			

**Table 3:** Univariable and multivariable analysis for the OS.

Overall Survival						
	Univariable Analysis			Multivariable Analysis		
	*p*-Value	HR	95% CI	*p*-Value	HR	95% CI
**Primary Tumor**						
SUVmax above median	0.510	0.78	0.38–1.62			
MTV above median	0.706	0.87	0.42–1.80			
TLG above median	0.697	0.87	0.42–1.79			
**Metastases**						
SUVmax above median	0.556	1.27	0.57–2.82			
MTV (40%) above median	0.006	3.94	1.50–10.39			
TLG (40%) above median	0.006	3.88	1.47–10.23	0.024	3.12	1.16–8.35
**Sum of primary and metastases**						
MTV above median	0.929	0.97	0.44–2.12			
TLG above median	0.612	0.81	0.37–1.80			
**Clinical**						
ECOG (0 + 1 vs. 2)	<0.001	6.49	2.30–18.30	0.006	4.59	1.54–13.66
No. of metastases (1 vs. more)	0.318	1.53	0.66–3.54			
Systemic treatment (yes vs. no)	0.048	2.14	1.01–4.53			
Brain involvement (yes vs. no)	0.767	0.89	0.40–1.96			

## Data Availability

The data are available from the authors upon reasonable request.

## Supplementary Materials

The following supporting information can be downloaded at: https://www.mdpi.com/article/10.3390/cancers18121880/s1, Table S1: Table summarizing metabolic parameters of the primary tumors and metastases.

## Abbreviations

The following abbreviations are used in this manuscript:

ACC	adenocarcinoma
CRT	radiochemotherapy
^18^F -FDG PET/CT	^18^F-fluorodeoxyglucose positron emission tomography/computed tomography
LAT	local ablative therapies
LCC	large-cell carcinoma
MTV	metabolic tumor volume
N/A	not applicable
NOS	not otherwise specified
NSCLC	non-small cell lung cancer
OMD	oligometastatic disease
OMD NSCLC	oligometastatic non-small cell lung cancer
OS	overall survival
PFS	progression-free survival
SABR	stereotactic ablative radiotherapy
SBRT	Stereotactic body radiotherapy
SCC	squamous-cell carcinoma
TLG	total lesion glycolysis

## References

[1] Hendriks L., Kerr K., Menis J., Mok T., Nestle U., Passaro A., Peters S., Planchard D., Smit E., Solomon B. (2023). Non-oncogene-addicted metastatic non-small-cell lung cancer: ESMO Clinical Practice Guideline for diagnosis, treatment and follow-up. Ann. Oncol..

[2] Łazar-Poniatowska M., Bandura A., Dziadziuszko R., Jassem J. (2021). Concurrent chemoradiotherapy for stage III non-small-cell lung cancer: Recent progress and future perspectives (a narrative review). Transl. Lung Cancer Res..

[3] Hellman S., Weichselbaum R.R. (1995). Oligometastases. J. Clin. Oncol..

[4] Weichselbaum R.R., Hellman S. (2011). Oligometastases revisited. Nat. Rev. Clin. Oncol..

[5] Hendriks L.E., Dooms C., Berghmans T., Novello S., Levy A., De Ruysscher D., Hasan B., Levra M.G., Levra N.G., Besse B. (2019). Defining oligometastatic non-small cell lung cancer: A simulated multidisciplinary expert opinion. Eur. J. Cancer.

[6] Lievens Y., Guckenberger M., Gomez D., Hoyer M., Iyengar P., Kindts I., Romero A.M., Nevens D., Palma D., Park C. (2020). Defining oligometastatic disease from a radiation oncology perspective: An ESTRO-ASTRO consensus document. Radiother. Oncol..

[7] Dingemans A.-M.C., Hendriks L.E., Berghmans T., Levy A., Hasan B., Faivre-Finn C., Giaj-Levra M., Giaj-Levra N., Girard N., Greillier L. (2019). Definition of Synchronous Oligometastatic Non–Small Cell Lung Cancer—A Consensus Report. J. Thorac. Oncol..

[8] Guckenberger M., Lievens Y., Bouma A.B., Collette L., Dekker A., Desouza N.M., Dingemans A.-M.C., Fournier B., Hurkmans C., Lecouvet F.E. (2020). Characterisation and classification of oligometastatic disease: A European Society for Radiotherapy and Oncology and European Organisation for Research and Treatment of Cancer consensus recommendation. Lancet Oncol..

[9] Gibas B.H., Teodorczyk J., Cytawa W., Bandura A. (2025). Long-term Disease Control in Lung Adenocarcinoma with Recurrent Oligometastases: PET-guided Management. Indian J. Nucl. Med..

[10] Bandura A., Dinh P.T., Wrona A., Konopa K., Dziadziuszko R. (2025). Treatment of oligometastatic non-small cell lung cancer with radiotherapy—Single-centre experience. Wspolczesna Onkol. Oncol..

[11] Guberina M., Pöttgen C., Guberina N., Hoffmann C., Wiesweg M., Richlitzki C., Metzenmacher M., Aigner C., Bölükbas S., Gauler T. (2024). Long-Term Survival in Patients with Oligometastatic Non-Small Cell Lung Cancer by a Multimodality Treatment—Comparison with Stage III Disease. Cancers.

[12] van Tinteren H., Hoekstra O.S., Smit E.F., Bergh J.H.v.D., Schreurs A.J., Stallaert R.A., van Velthoven P.C., Comans E.F., Diepenhorst F.W., Verboom P. (2002). Effectiveness of positron emission tomography in the preoperative assessment of patients with suspected non-small-cell lung cancer: The PLUS multicentre randomised trial. Lancet.

[13] Fischer B., Lassen U., Mortensen J., Larsen S., Loft A., Bertelsen A., Ravn J., Clementsen P., Høgholm A., Larsen K. (2009). Preoperative Staging of Lung Cancer with Combined PET–CT. N. Engl. J. Med..

[14] de Geus-Oei L., van der Heijden H.F., Corstens F.H., Oyen W.J. (2007). Predictive and prognostic value of FDG-PET in nonsmall-cell lung cancer. Cancer.

[15] Du B., Wang S., Cui Y., Liu G., Li X., Li Y. (2021). Can^18^F-FDG PET/CT predict EGFR status in patients with non-small cell lung cancer? A systematic review and meta-analysis. BMJ Open.

[16] Liang C., Zheng M., Zou H., Han Y., Zhan Y., Xing Y., Liu C., Zuo C., Zou J. (2024). Deep learning-based image analysis predicts PD-L1 status from 18F-FDG PET/CT images in non-small-cell lung cancer. Front. Oncol..

[17] Seol H.Y., Kim Y.S., Kim S. (2020). Predictive value of 18F-fluorodeoxyglucose positron emission tomography/computed tomography for PD-L1 expression in non-small cell lung cancer: A systematic review and meta-analysis. Thorac. Cancer.

[18] Shao X., Ge X., Gao J., Niu R., Shi Y., Shao X., Jiang Z., Li R., Wang Y. (2024). Transfer learning–based PET/CT three-dimensional convolutional neural network fusion of image and clinical information for prediction of EGFR mutation in lung adenocarcinoma. BMC Med. Imaging.

[19] Tomasik B., Jąkalski M., Bieńkowski M., Teodorczyk J., Sobocki B.K., Stawiski K., Burzynski J., Romanowicz G., Dziadziuszko R., Cytawa W. (2026). Non-invasive assessment of PD-L1 status and histology in non-small cell lung cancer using 18F-FDG PET/CT radiomics. J. Transl. Med..

[20] Im H.J., Bradshaw T., Solaiyappan M., Cho S.Y. (2018). Current Methods to Define Metabolic Tumor Volume in Positron Emission Tomography: Which One is Better?. Nucl. Med. Mol. Imaging..

[21] Vanhove K., Mesotten L., Heylen M., Derwael R., Louis E., Adriaensens P., Thomeer M., Boellaard R. (2018). Prognostic value of total lesion glycolysis and metabolic active tumor volume in non-small cell lung cancer. Cancer Treat. Res. Commun..

[22] Ogliari F.R., Jongbloed M., Vaes R.D.W., Houben R.M.A., Bartolomeo V., Borne B.E.E.M.v.D., Degens J., Huijs J.W.J., Pitz C., Steens M. (2025). Association of metabolic tumour volume (MTV) and total lesion glycolysis (TLG) with survival in patients with oligometastatic non-small-cell lung cancer treated with immunotherapy: A multicentre retrospective study. Eur. J. Nucl. Med..

[23] Torresan S., Costa J., Zanchetta C., De Marchi L., Rizzato S., Cortiula F. (2025). Oligometastatic NSCLC: Current Perspectives and Future Challenges. Curr. Oncol..

[24] Zer A., Ahn M.-J., Barlesi F., Bubendorf L., De Ruysscher D., Garrido P., Gautschi O., Hendriks L., Jänne P., Kerr K. (2025). Early and locally advanced non-small-cell lung cancer: ESMO Clinical Practice Guideline for diagnosis, treatment and follow-up. Ann. Oncol..

[25] Iyengar P., All S., Berry M.F., Boike T.P., Bradfield L., Dingemans A.-M.C., Feldman J., Gomez D.R., Hesketh P.J., Jabbour S.K. (2023). Treatment of Oligometastatic Non-Small Cell Lung Cancer: An ASTRO/ESTRO Clinical Practice Guideline. Pract. Radiat. Oncol..

[26] Brose A., Miederer I., König J., Gkika E., Sahlmann J., Schimek-Jasch T., Schreckenberger M., Nestle U., Kappes J., Miederer M. (2024). Prognostic value of metabolic tumor volume on [18F]FDG PET/CT in addition to the TNM classification system of locally advanced non-small cell lung cancer. Cancer Imaging.

[27] Bazan J.G., Duan F., Snyder B.S., Horng D., Graves E.E., Siegel B.A., Machtay M., Loo B.W. (2017). Metabolic tumor volume predicts overall survival and local control in patients with stage III non-small cell lung cancer treated in ACRIN 6668/RTOG 0235. Eur. J. Nucl. Med. Mol. Imaging.

[28] Deshpande S.R., Podder T.K., Grubb W., Zhang Y., Zheng Y., Towe C., Linden P., Avril N., Biswas T. (2023). Pretreatment and Posttreatment Tumor Metabolic Activity Assessed by FDG-PET/CT as Predictors of Tumor Recurrence and Survival Outcomes in Early-Stage Non-Small Cell Lung Cancer Treated With Stereotactic Body Radiation Therapy. Adv. Radiat. Oncol..

[29] Dong M., Liu J., Sun X., Xing L. (2017). Prognositc significance of SUV_max_ on pretreatment ^18^F-FDG PET/CT in early-stage non-small cell lung cancer treated with stereotactic body radiotherapy: A meta-analysis. J. Med. Imaging Radiat. Oncol..

[30] Dosani M., Yang R., McLay M., Wilson D., Liu M., Yong-Hing C.J., Hamm J., Lund C.R., Olson R., Schellenberg D. (2019). Metabolic Tumour Volume Is Prognostic in Patients with Non-Small-Cell Lung Cancer Treated with Stereotactic Ablative Radiotherapy. Curr. Oncol..

[31] Tosi D., Pieropan S., Cattoni M., Bonitta G., Franzi S., Mendogni P., Imperatori A., Rotolo N., Castellani M., Cuzzocrea M. (2021). Prognostic Value of 18F-FDG PET/CT Metabolic Parameters in Surgically Treated Stage I Lung Adenocarcinoma Patients. Clin. Nucl. Med..

[32] Ling T., Zhang L., Peng R., Yue C., Huang L. (2022). Prognostic value of 18F-FDG PET/CT in patients with advanced or metastatic non-small-cell lung cancer treated with immune checkpoint inhibitors: A systematic review and meta-analysis. Front. Immunol..

[33] Huang M., Zou Y., Wang W., Li Q., Tian R. (2024). The role of baseline ^18^F-FDG PET/CT for survival prognosis in NSCLC patients undergoing immunotherapy: A systematic review and meta-analysis. Ther. Adv. Med. Oncol..

[34] Chin A.L., Kumar K.A., Guo H.H., Maxim P.G., Wakelee H., Neal J.W., Diehn M., Loo B.W., Gensheimer M.F. (2018). Prognostic Value of Pretreatment FDG-PET Parameters in High-dose Image-guided Radiotherapy for Oligometastatic Non–Small-cell Lung Cancer. Clin. Lung Cancer.

[35] Greco C., Pares O., Pimentel N., Louro V., Morales J., Nunes B., Castanheira J., Oliveira C., Silva A., Vaz S. (2019). Phenotype-Oriented Ablation of Oligometastatic Cancer with Single Dose Radiation Therapy. Int. J. Radiat. Oncol..

[36] Greco C., Pares O., Pimentel N., Louro V., Morales J., Nunes B., Antunes I., Vasconcelos A.L., Kociolek J., Castanheira J. (2021). Positron Emission Tomography–Derived Metrics Predict the Probability of Local Relapse After Oligometastasis-Directed Ablative Radiation Therapy. Adv. Radiat. Oncol..

[37] Mazzola R., Fiorentino A., Di Paola G., Levra N.G., Ricchetti F., Fersino S., Tebano U., Pasetto S., Ruggieri R., Salgarello M. (2017). Stereotactic Ablative Radiation Therapy for Lung Oligometastases: Predictive Parameters of Early Response by 18 FDG-PET/CT. J. Thorac. Oncol..

[38] Rahmim A., Bak-Fredslund K.P., Ashrafinia S., Lu L., Schmidtlein C.R., Subramaniam R.M., Morsing A., Keiding S., Horsager J., Munk O.L. (2019). Prognostic modeling for patients with colorectal liver metastases incorporating FDG PET radiomic features. Eur. J. Radiol..

[39] Willmann J., Badra E.V., Adilovic S., Christ S.M., Ahmadsei M., Mayinger M., Tanadini-Lang S., Guckenberger M., Andratschke N. (2022). Distant Metastasis Velocity as a Novel Prognostic Score for Overall Survival After Disease Progression Following Stereotactic Body Radiation Therapy for Oligometastatic Disease. Int. J. Radiat. Oncol..

[40] Chang J.S., Dunne E.M., Baker S., Liu M. (2024). Beyond lesion count: Emphasizing disease pace in oligometastatic management. Radiother. Oncol..

[41] Klement R.J., Sweeney R.A. (2023). Metabolic factors associated with the prognosis of oligometastatic patients treated with stereotactic body radiotherapy. Cancer Metastasis Rev..

[42] Lugano R., Ramachandran M., Dimberg A. (2020). Tumor angiogenesis: Causes, consequences, challenges and opportunities. Cell. Mol. Life Sci..

[43] Hanahan D. (2022). Hallmarks of Cancer: New Dimensions. Cancer Discov..

[44] Lu J. (2019). The Warburg metabolism fuels tumor metastasis. Cancer Metastasis Rev..

[45] Vanhove K., Thomeer M., Derveaux E., Shkedy Z., Owokotomo O.E., Adriaensens P., Mesotten L. (2019). Correlations between the metabolic profile and 18F-FDG-Positron Emission Tomography-Computed Tomography parameters reveal the complexity of the metabolic reprogramming within lung cancer patients. Sci. Rep..

[46] Avella D.M., Manjunath Y., Singh A., Deroche C.B., Kimchi E.T., Staveley-O’cArroll K.F., Mitchem J.B., Kwon E., Li G., Kaifi J.T. (2020). 18F-FDG PET/CT total lesion glycolysis is associated with circulating tumor cell counts in patients with stage I to IIIA non-small cell lung cancer. Transl. Lung Cancer Res..

[47] Shang J., You H., Dong C., Li Y., Cheng Y., Tang Y., Guo B., Gong J., Ling X., Xu H. (2022). Predictive value of baseline metabolic tumor burden on 18F-FDG PET/CT for brain metastases in patients with locally advanced non-small-cell lung cancer. Front. Oncol..

