# Supplementary material for: Prognostic Role of 18F-FDG PET/CT in Oligometastatic Non-Small Cell Lung Cancer: Preliminary Results from Single Center
Source: Cancers (Basel). 2026 Jun 9;18(12):1880. doi: 10.3390/cancers18121880 (PMC13297079; doi:10.3390/cancers18121880)
Supplement: Supplementary file 1 [file cancers-18-01880-s001.zip › cancers-4306094-supplementary.pdf]

cancers

MDPI

Table S1. Table summarizing metabolic parameters of the primary tumors and metastases.

	**Median value (IQR)**
Primary tumor SUVmax	16.07 SUV-bw (12.64–20.48)
Primary tumor MTV	10.52 cm^3 (4.11–27.70)
Primary tumor TLG	83.59 SUV × cm^3 (34.14–293.58)
Metastases SUVmax	8.61 SUV-bw (5.70–15.94)
Metastases MTV	2.19 cm^3 (1.05–7.65)
Metastases TLG	11.01 SUV × cm^3 (4.90–44.82)
